# Efficacy and recovery of remimazolam versus midazolam in sedated upper gastrointestinal endoscopy: a multicenter randomized controlled trial in Japan (RECOVER Study)

**DOI:** 10.1007/s00535-025-02324-x

**Published:** 2025-11-17

**Authors:** Daisuke Yamaguchi, Ryoji Ichijima, Hisatomo Ikehara, Yosuke Minoda, Mitsuru Esaki, Ayako Takamori, Akiyoshi Yoh, Moeko Shirouzu, Kento Sadashima, Yutaro Fujimura, Takuya Shimamura, Hironobu Takedomi, Takashi Akutagawa, Nanae Tsuruoka, Yasuhisa Sakata, Takuya Wada, Chika Kusano, Ryo Shimoda, Motohiro Esaki

**Affiliations:** 1 Division of Gastroenterology, Department of Internal Medicine, Faculty of Medicine, Saga University, Saga, 849-8501 Japan; 2 Department of Gastroenterology, Saiseikai Kawaguchi General Hospital, Kawaguchi, Japan; 3 Endoscopy Division, National Cancer Center Hospital, Tokyo, Japan; 4 Department of Gastroenterology, Internal Medicine, Kitasato University School of Medicine, Sagamihara, Japan; 5 Department of Medicine and Bioregulatory Science, Graduate School of Medical Sciences, Kyushu University, Fukuoka, Japan; 6 Clinical Research Center, Saga University Hospital, Saga, Japan; 7 Clinical Research Center in Hiroshima, Hiroshima University Hospital, Hiroshima, Japan; 8 Department of Endoscopic Diagnostics and Therapeutics, Saga University Hospital, Saga, Japan

**Keywords:** Sedation, Upper gastrointestinal endoscopy, Remimazolam, Midazolam, MOAA/S score

## Abstract

**Objectives:**

Sedation is increasingly essential for gastrointestinal endoscopy. Remimazolam, an ultra-short-acting benzodiazepine, has a shorter pharmacokinetic half-life than midazolam. The aim of this study was to determine whether remimazolam provides superior procedural sedation in Japanese patients.

**Methods:**

The cohort of this prospective, multicenter, randomized, single-blind controlled trial comprised adults (18–80 years) scheduled for sedated upper gastrointestinal endoscopy. Participants were randomized to remimazolam and midazolam groups. The primary outcome was the proportion of ambulatory patients 5 min after endoscopy. Secondary outcomes were successful pre-procedure sedation (Modified Observer’s Assessment of Alertness/Sedation ≤ 4), dose of sedative to achieve sedation, time to ambulation, and adverse events.

**Results:**

From October 2024 to January 2025, 40 patients were enrolled. After excluding two outliers 38 were analyzed (remimazolam, n = 20; midazolam, n = 18). Ambulation at 5 min occurred in 85.0% (17/20) of the remimazolam versus 0.0% (0/18) of the midazolam group (*p* < 0.0001). Mean time from procedure end to walking was 4.25 min (range 0.0–10.0) for remimazolam and 35.56 min (10.0–60.0) for midazolam (*p* < 0.0001). Pre-procedure sedation was successful (MOAA/S ≤ 4) in 100% of both groups. Mean doses to achieve sedation were 4.30 mg (3.0–7.0) for remimazolam and 3.11 mg (2.0–5.0) for midazolam (*p* = 0.003). Hypoxemia occurred in 5.0% of the remimazolam and 33.3% of the midazolam group (*p* = 0.038).

**Conclusions:**

In upper gastrointestinal endoscopy, remimazolam achieved markedly faster recovery and a lower incidence of hypoxemia than midazolam. Rates of achieving target sedation were equivalent. These findings indicate remimazolam is an effective and potentially safer sedative option for Japanese patients undergoing endoscopy.

*Trial registration*: This research was registered with the Japan Registry of Clinical Trials (trial number jRCTs071240062) on September 26, 2024.

**Supplementary Information:**

The online version contains supplementary material available at 10.1007/s00535-025-02324-x.

## Introduction

Demand for sedation during gastrointestinal (GI) endoscopy is continuing to increase, to the extent that it is now considered an essential component of routine clinical care [1]. The Japanese Society of Gastrointestinal Endoscopy’s “Guidelines for Sedation in Gastrointestinal Endoscopy (Second Edition)” [2] highlights that sedation improves patient acceptability and satisfaction for both upper GI endoscopy and colonoscopy, which in turn enhances diagnostic and therapeutic outcomes. As a result, the proportion of sedation endoscopy has been increasing in Japan [2–6].

However, fewer sedatives are covered in Japan by national health insurance than in many Western countries, leading to off-label reliance on benzodiazepines. Midazolam, a benzodiazepine, remains the most commonly used agent in daily practice [2, 7–10]. Its relatively long elimination half-life means there may be residual effects after the examination or intervention, delaying departure from the endoscopy suite and prolonging recovery room stays [11, 12]. Given the limited number of endoscopy recovery beds, these delays can constrain broader use of sedatives.

Remimazolam, an ultra-short-acting benzodiazepine approved by the US Food and Drug Administration, was added to Japan’s insurance coverage for endoscopic sedation in June 2025. Considering its shorter pharmacokinetic half-life than midazolam, it was anticipated that remimazolam would substantially shorten both time to recover consciousness after endoscopy and time to discharge from the recovery area, making it a promising option for endoscopic sedation [13–17]. We have previously reported its utility and safety in a randomized, placebo-controlled trial in Japanese patients [18, 19]. The safety and efficacy of remimazolam in endoscopic examinations have also been demonstrated in several overseas studies [20–25]. Furthermore, some retrospective propensity score matching studies comparing remimazolam with midazolam have been reported from Japan [26, 27]. However, to the best of our knowledge, no prospective randomized head-to-head comparisons between remimazolam and midazolam in endoscopic procedures conducted in Japan have been published to date.

We thus conducted this REmimazolam COmpared to Versed (midazolam) for Endoscopic Recovery (RECOVER) study with the aim of directly comparing the sedative profiles of midazolam and remimazolam in Japanese patients undergoing GI endoscopy and thus clarify the clinical value of remimazolam in real-world practice.

## Methods

### Study design

The RECOVER study was a prospective, multicenter, randomized, single-blind, active-controlled trial conducted at four Japanese hospitals (Saga University Hospital, Saiseikai Kawaguchi General Hospital, Kitasato University Hospital, and Kyushu University Hospital). Adults scheduled for outpatient GI endoscopy under sedation were enrolled after providing written informed consent. Randomization was centralized via the UMIN INDICE cloud system using a minimization algorithm with the factors of site, age (18–69 vs. 70–80 years), and sex. Patients were blinded to the allocated sedative agent whereas investigators were not blinded for safety reasons, creating a single-masked design. Screening occurred within 28 days before endoscopy and follow-up ended the day after endoscopy. The trial was prospectively registered in the Japan Registry of Clinical Trials (jRCT; no. jRCTs071240062; registered 26 September 2024; https://jrct.niph.go.jp/). This study was conducted in accordance with the tenets of the Declaration of Helsinki and the guidelines of the Consolidated Standards of Reporting Trials.

### Inclusion and exclusion criteria

Inclusion criteria comprised: (1) written informed consent; (2) Japanese adult aged 18–80 years; and (3) scheduled upper GI endoscopy or colonoscopy with sedation. Exclusion criteria included: planned therapeutic endoscopy (polypectomy, endoscopic mucosal resection, endoscopic submucosal dissection); prior upper/lower GI surgery; dialysis; daily alcohol intake ≥ 60 g; severe impairment of hepatic function; American Society of Anesthesiologists physical status (ASA-PS) III–V; inability to ambulate independently; regular use of benzodiazepines, analgesics, or central nervous system depressants; use of any sedative within the previous 4 weeks; acute angle-closure glaucoma; myasthenia gravis; pregnancy or breastfeeding; hypersensitivity to benzodiazepines or flumazenil or other contraindications; and any other condition that the investigator considered warranted exclusion.

### Study procedure and sedative administration

Participants were allocated in a 1:1 ratio to remimazolam and midazolam groups. The dosages of sedative were those established in previous studies [16, 18, 19]. Namely, remimazolam (50-mg vial reconstituted with 50 mL saline; 1 mg/mL) was given as an initial 3-mg intravenous bolus followed by 1-mg supplemental boluses as needed. Midazolam (10 mg/2 mL diluted with 8 mL saline; 1 mg/mL) was given as an initial 2-mg intravenous bolus followed by 1-mg supplemental boluses as needed. All boluses were administered over ≥ 15 s at intervals of ≥ 2 min, not exceeding 10 mg (pre- and intra-procedural combined). Details of the sedative administration method are shown in Supplemental Fig. 1.

The level of sedation was evaluated using the Modified Observer’s Assessment of Alertness/Sedation (MOAA/S) score [28]. The appropriate level of sedation for endoscopy is moderate sedation, requiring the administration of sedatives to achieve a MOAA/S score of 4, corresponding to a “Lethargic response to name spoken in normal tone” or lower. Details of the MOAA/S score are shown in Supplemental Fig. 2. MOAA/S scores were determined ≥ 2 min after each bolus. Endoscopy began once adequate sedation (MOAA/S score ≤ 4) had been achieved. If the MOAA/S score remained 5 after the administration of the maximum total dose, the sedative was deemed ineffective and endoscopy proceeded without further study sedative (“sedation failure”). During endoscopy, the MOAA/S score was checked every 2 min. If signs of arousal (MOAA/S score of 5 or clinically relevant movement) were observed, the patient received additional sedatives at intervals of at least 2 min from the previous dose initiation until the end of the endoscopy. Maintenance doses were then administered based on the same algorithm, not exceeding a total of 10 mg.

After scope withdrawal, MOAA/S scores were recorded at 0, 5, 10, 20, and 30 min and every 10 min thereafter until the MOAA/S score reached 5. At this point, a standardized gait test was performed: the ability to walk 5 m in a straight line without staggering and the time from the end of the procedure to ambulation were recorded. If the MOAA/S score was not 5 at 30 min, assessments continued at 10-min intervals until recovery. Patients remained in the recovery room for at least 30 min after the procedure, leaving the endoscopy suite only after confirming an MOAA/S score of 5 and adequate ambulation. For safety reasons, the patient was accompanied home by family or friends and was prohibited from driving a car on that or the following day. Satisfaction and interval events were assessed by next-day telephone follow-up.

### Study endpoints

The primary endpoint was the proportion of participants able to ambulate independently 5 min after the end of endoscopy (“effective sedation”). We determined provisions similar to those for successful sedation established in a physician-initiated Phase III clinical trial conducted in Japan among patients undergoing gastrointestinal endoscopy [18]. Secondary endpoints comprised: (1) successful pre-procedure sedation (MOAA/S score ≤ 4); (2) dose required to achieve pre-procedure sedation; (3) total dose required to complete endoscopy; (4) time from first dose to commencing endoscopy (MOAA/S score ≤ 4); (5) time from end of procedure to decision to start walking (MOAA/S score = 5); (6) total time from end of procedure to completion of walking (including rest); (7) patient satisfaction (5-point scale) on discharge and as ascertained by phone the following day; and (8) endoscopist satisfaction (5-point scale).

### Definitions

Two physicians were deployed in the endoscopy room: the physician who performed the endoscopic examination determined the MOAA/S score and the other physician was responsible for assessing and intervening when unexpected adverse events occurred. Japan Gastroenterological Endoscopy Society-certified 13 specialists performed all procedures. All participating physicians held current American Heart Association–accredited Basic Life Support certification. Completion of upper GI endoscopy required adequate inspection of the esophagus, stomach (including retroflex view of the body), and duodenum to the descending portion. Loss of consciousness was defined as a MOAA/S score ≤ 1 at two consecutive time points. Oxygen (2–5 L/min) was administered if SpO₂ fell to < 94%. An airway was secured when the respiratory rate was < 5 breaths/min; if breathing did not recover, bag-valve-mask ventilation was initiated. Flumazenil was administered only for urgent reversal. The severity of adverse events was graded according to the Common Terminology Criteria for Adverse Events, version 5.0. After endoscopy, patients and endoscopists were asked to grade overall satisfaction (grade 1–5; best scored as 5) encompassing sedation effects, recovery effects, and adverse events.

### Sample size

After referencing previous studies [16, 18, 19] and assuming ambulation at 5 min of 80% with remimazolam and 35% with midazolam (two-sided α = 0.05, power = 80%), we concluded that 36 patients (18 per group) were required. Allowing for approximately 10% attrition and balanced accrual across the four participating sites, the planned enrollment was 40 patients (20 per group). No interim analyses were planned.

### Statistical analysis

Continuous variables are expressed as median (interquartile range) and were compared using the Wilcoxon rank-sum test; categorical variables are expressed as counts (percentages) and were compared using Fisher’s exact test. Questionnaire responses are reported as the proportion of 5-point ratings. Patient and endoscopist questionnaires were analyzed separately using the Wilcoxon rank-sum test. Two-sided p < 0.05 was taken to denote statistical significance. Analyses were performed with JMP, version 17.0.0 (SAS Institute, Cary, NC, USA).

## Results

### Baseline characteristics

**Fig. 1:**
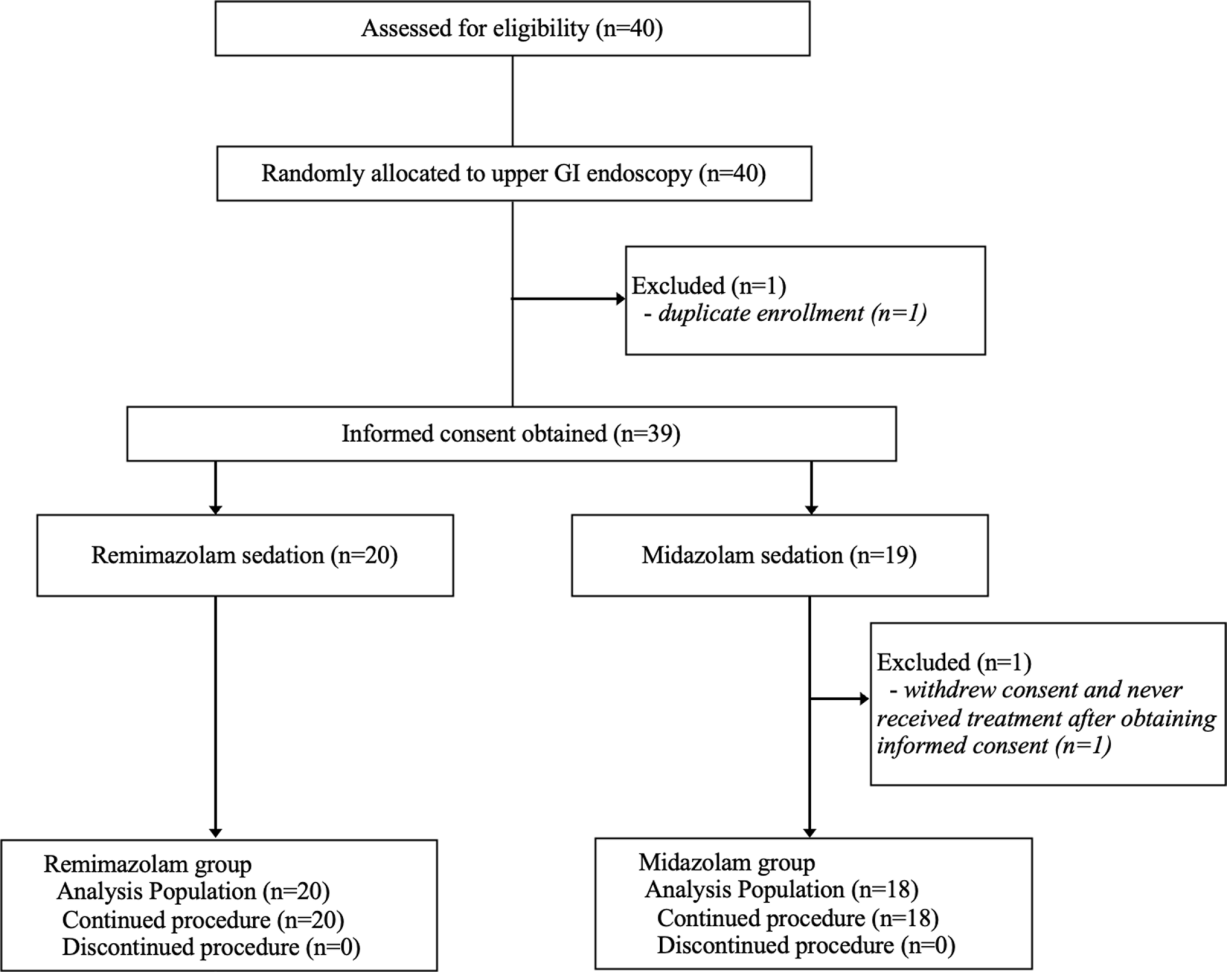
Flow diagram of the study

Figure 1 is the patient flowchart. Data for 38 patients (two outliers were excluded) enrolled from October 2024 to January 2025 were analyzed (remimazolam group, n = 20; midazolam group, n = 18). In Table 1, baseline characteristics and comorbidities are compared between the two groups. Compared variables were well balanced with no significant between-group differences in age (61.9 ± 14.0 vs. 59.2 ± 17.3 years, *p* = 0.594), sex (male 50.0% vs. 55.6%, *p* = 0.732), body mass index (22.2 ± 2.7 vs. 23.5 ± 2.9 kg/m^2^, *p* = 0.140), ASA-PS class (I/II: 40.0/60.0% vs. 33.3/66.7%, p = 0.671), smoking history (20.0% vs. 38.9%, *p* = 0.200), or major comorbidities.

**Table 1:** Baseline characteristics

	Remimazolam group	Midazolam group	p*-*value
Number of patients (N)	20	18	
Age (years)	61.9 ± 14.0	59.2 ± 17.3	0.594
Sex, males	10 (50.0%)	10 (55.6%)	0.732
Height (cm)	162.7 ± 10.9	163.0 ± 9.6	0.926
Weight (kg)	59.2 ± 12.6	62.6 ± 9.9	0.369
BMI (kg/m2)	22.2 ± 2.7	23.5 ± 2.9	0.140
ASA-PS, I	8 (40.0%)	6 (33.3%)	0.671
ASA-PS, II	12 (60.0%)	12 (66.7%)	
Smoking history	4 (20.0%)	7 (38.9%)	0.200
Comorbidities			
Hypertension	6 (30.0%)	6 (33.3%)	0.825
Diabetes mellitus	3 (15.0%)	4 (22.2%)	0.566
Dyslipidemia	2 (10.0%)	5 (27.8%)	0.222
Mild lung disease	2 (10.0%)	1 (5.6%)	0.612
GI malignancy	5 (25.0%)	3 (16.7%)	0.697

Results are presented as the number of patients or mean ± standard deviation

BMI: body mass index, ASA-PS: American Society of Anesthesiologists physical status GI: gastrointestinal

### Sedation rate (primary endpoint)

Five minutes after ending upper GI endoscopy, 85.0% (17/20) of patients in the remimazolam group versus 0.0% (0/18) in the midazolam group (χ^2^ = 27.7, p < 0.0001) were able to ambulate. The 95% CIs were 62.1%–96.8% for the remimazolam group and 0.0%–18.5% for the midazolam group (Table 2).

**Table 2:** Percentage of study patients who achieved ambulation 5 min after endoscopy

Group	n	Ambulation at 5 min	95% CI			χ^2^	p-value
Remimazolam	20	17 (85.0%)	62.1%	–	96.8%	27.7	< 0.0001
Midazolam	18	0 (0.0%)	0.0%	–	18.5%		

This difference is statistically significant (χ^2^ = 27.7, *p* < 0.0001)

### Assessment of efficacy of sedation

Sedation was achieved before upper GI endoscopy (MOAA/S score ≤ 4) in all patients (100%) in both groups. Times required to achieve sedation and to become ambulatory after endoscopy are presented in Table 3. There was no significant difference between the two groups (3.90 ± 2.63 vs. 3.89 ± 1.75 min, *p* = 0.988) in the time required to start sedation for endoscopy. The procedure time was equivalent between the two groups (6.50 ± 2.33 vs. 6.56 ± 2.55 min, p = 0.944). Recovery was markedly faster in the remimazolam group than in the midazolam group: times from ending endoscopy to MOAA/S score = 5 being 2.00 ± 3.40 vs. 27.78 ± 14.97 min (p < 0.0001), and times from ending endoscopy to becoming ambulatory 4.25 ± 3.35 vs. 35.56 ± 12.47 min, respectively (p < 0.0001).

**Table 3:** Times to achieving sedation and to achieving ambulation after endoscopy

	Group	n	Mean	SD	Min–Max	Mean difference	[ 95% CI]	p-value
Time to start endoscopy start (min)	Remimazolam	20	3.90	2.63	2.0–10.0	0.01	[-1.48, 1.50]	0.988
	Midazolam	18	3.89	1.75	2.0–8.0			
Procedure time (min)	Remimazolam	20	6.50	2.33	4.0–10.0	-0.06	[− 1.66, 1.55]	0.944
	Midazolam	18	6.56	2.55	4.0–14.0			
Time from endoscopy end to MOAA/S score = 5 (min)	Remimazolam	20	2.00	3.40	0–10.0	− 25.78	[-32.75, -18.81]	< .0001
	Midazolam	18	27.78	14.97	5.0–50.0			
Total time from ending endoscopy to achieving ambulation (min)	Remimazolam	20	4.25	3.35	0–10.0	− 31.31	[-37.18, -25.43]	< .0001
	Midazolam	18	35.56	12.47	10.0–60.0			

Table 4 shows the dosage of sedative required for upper GI endoscopy. The doses to achieve/maintain sedation differed between the study groups: pre-endoscopy 3.95 ± 1.32 mg vs. 2.94 ± 0.87 mg (p = 0.010), during endoscopy 0.35 ± 0.59 mg vs. 0.17 ± 0.38 mg (p = 0.268), and total 4.30 ± 1.34 mg vs. 3.11 ± 0.83 mg (p = 0.003) for remimazolam and midazolam, respectively. The proportion of patients who completed the examination with the initial dose alone showed no significant difference between the two groups (remimazolam group 40.0% vs midazolam group 22.2%, p = 0.307). No significant difference was found in baseline characteristics between patients who required an additional bolus dose and those who did not in the remimazolam and midazolam groups.

**Table 4:** Sedative dosage required for upper GI endoscopy

	Group	n	Mean	SD	Min–Max	Mean difference	[ 95% CI]	p-value
Pre-endoscopy dosage	Remimazolam	20	3.95	1.32	3.0–7.0	1.01	[0.26, 1.75]	0.010
(mg)	Midazolam	18	2.94	0.87	2.0–5.0			
During endoscopy dosage	Remimazolam	20	0.35	0.59	0–2.0	0.18	[-0.15, 0.51]	0.268
(mg)	Midazolam	18	0.17	0.38	0–1.0			
Total dosage	Remimazolam	20	4.30	1.34	3.0–7.0	1.19	[0.44, 1.93]	0.003
(mg)	Midazolam	18	3.11	0.83	2.0–5.0			

GI: gastrointestinal

### Safety assessments of sedation

Adverse events are summarized in Table 5. Oxygen was required in 1/20 patients (5.0%) in the remimazolam group and 6/18 (33.3%) in the midazolam group (p = 0.038). Hypertension occurred in 1/20 (5.0%) and 1/18 (5.6%) patients, respectively; no hypotension or headache occurred in either group. No patient in either group required administration of flumazenil or bag-valve-mask ventilation during endoscopy. No grade III–V adverse events were detected.

**Table 5:** Adverse events

	Remimazolam group (n = 20)	Midazolam group (n = 18)	*p* value
*Adverse event grade (I/II/III–V)*			
Oxygen administration	1 (5.0%) (0/1/0)	6 (33.3%) (5/1/0)	0.038
Hypertension	1 (5.0%) (1/0/0)	1 (5.6%) (1/0/0)	1.000
Hypotension	0 (0.0%) (0/0/0)	0 (0.0%) (0/0/0)	1.000
Flumazenil administration	0 (0.0%) (0/0/0)	0 (0.0%) (0/0/0)	1.000
Manual ventilation	0 (0.0%) (0/0/0)	0 (0.0%) (0/0/0)	1.000
Headache	0 (0.0%) (0/0/0)	0 (0.0%) (0/0/0)	1.000

### Patients’ and endoscopists’ assessments

Table 6 shows the patients’ and endoscopists’ assessments. Patient satisfaction (5-point scale) was high in both groups (4.70 ± 0.73 vs. 4.33 ± 0.77; p = 0.141); this difference is not significant. Endoscopist satisfaction was significantly higher for the remimazolam group (4.80 ± 0.52 vs. 3.61 ± 1.09; p = 0.0001).

**Table 6:** Patients’ and endoscopists’ assessments

	Group	n	Mean	SD	Min–Max	Mean difference	[ 95% CI]	p-value
Patients’ assessment	Remimazolam	20	4.70	0.73	3.0–5.0	0.37	[− 0.13, 0.86]	0.141
(grade 1–5)	Midazolam	18	4.33	0.77	3.0–5.0			
Endoscopists’ assessment	Remimazolam	20	4.80	0.52	3.0–5.0	1.19	[0.63, 1.74]	0.0001
(grade 1–5)	Midazolam	18	3.61	1.09	1.0–5.0			

## Discussion

To our knowledge, this RECOVER study is the first randomized, multicenter comparison of remimazolam and midazolam for upper GI endoscopy in Japan. Previous comparative studies from Japan were retrospective and propensity-matched rather than randomized [26, 27], highlighting the incremental value of the present trial’s design and endpoints. Remimazolam was approved in Japan for sedation for gastrointestinal endoscopic procedures on 24 June 2025 and added to the National Health Insurance Drug Price List on 14 August 2025, thereby enabling its use under Japan’s public health insurance system. This approval made a direct, head-to-head comparison with the long-standing standard sedative, midazolam, of considerable clinical relevance.

We previously demonstrated the safety of remimazolam in Phase III trials [18]. In the present study, we aimed to assess the recovery efficacy of remimazolam compared to midazolam that could be assumed as the most remarkable advantage of this sedative. We thus set the primary endpoint as “ambulation after 5 min” to evaluate recovery efficacy following endoscopy as had been set in the previous study [18]. This endpoint is important because quick arousal after sedative endoscopy can improve safe and smooth management of the recovery room. We also assessed sedation success rates and the degree of recovery after endoscopy as well, that had not been assessed in the previous RCTs [13, 14, 23]. Furthermore, these endpoints were evaluated in patients without concomitant use of analgesics in the present study. Thus, the present study is considered the true comparative study of remimazolam and midazolam in sedative gastrointestinal endoscopy.

The primary endpoint, unassisted ambulation at 5 min, was met by 85% of patients receiving remimazolam versus 0% of those receiving midazolam. Most patients in the remimazolam group were able to walk within 5 min after endoscopy, which is consistent with the findings of our previous study [18]. The fact that no one in the midazolam group was able to walk 5 min after ending endoscopy clearly indicates that consciousness is regained more quickly with remimazolam. Remimazolam promotes rapid recovery of consciousness following upper gastrointestinal endoscopy and reduces the time to ambulation, conferring a clinical advantage.

The rate of successfully achieving pre-endoscopy sedation (MOAA/S score ≤ 4) was 100% in both groups. These results are consistent with those of overseas trials comparing remimazolam and midazolam, demonstrating a high success rate [13–15]. In the present study, indicators of recovery favored remimazolam: while the time required to achieve sedation before endoscopy did not differ significantly between the groups, the times from endoscope removal to MOAA/S score = 5 and from ending endoscopy to ambulation were significantly shorter in the remimazolam group. Notably, the average time to ambulation after endoscopy was 35.56 min in the midazolam group but only 4.25 min in the remimazolam group. Demand for sedation for upper GI endoscopy continues to grow because it reduces patient discomfort and improves endoscopist satisfaction; however, the use of sedation requires more recovery space and additional monitoring staff [2, 29]. Using remimazolam could ease these operational burdens. Accordingly, it is expected that remimazolam will increasingly be used for sedated endoscopy, including in outpatient settings.

Although the total dose of sedative was higher in the remimazolam group, this difference was attributable to having used the initial doses specified in a previous study [18], which were 3 mg for remimazolam and 2 mg for midazolam. The absence of differences in additional doses during endoscopy between the two groups indicates that it is feasible to use the same method for additional administration of remimazolam as has been used for midazolam. However, since remimazolam is a newly approved drug, higher cost is a main disadvantage. The average cost per patient was calculated to be JPY 331.1 for remimazolam and JPY 17.9 for midazolam in this study setting.

Our findings related to safety were generally reassuring. Remimazolam was associated with a lower frequency of supplemental oxygen and no patients required flumazenil or bag-valve mask ventilation, while pre-endoscopy, during endoscopy and total dosages corrected by molar mass were equivalent between the two groups (Supplementary Table 1). This result might be caused by the shorter duration of activity of remimazolam, probably caused by the difference in metabolic pathway, mode and affinity to benzodiazepine receptors compared to midazolam. Such a milder respiratory suppression of remimazolam could bring secure use for endoscopic staff. In addition, all patients in the remimazolam group could leave the endoscopy room 30 min after the endoscopy in this study. Although further investigation with a larger sample size is necessary to generalize this finding into all cases, the quick recovery effect of remimazolam could contribute to the safety issue of sedative endoscopy.

Patients’ satisfaction was high in both groups. The greater endoscopists’ satisfaction in the remimazolam group likely reflects the high success of endoscopic sedation, a smooth endoscopy workflow, and a low incidence of adverse events. Combined with the significant reduction in recovery time observed in this study, these factors could lead to shorter procedure and recovery room occupancy times, a reduction of staff burden, and more reliable improvement in endoscopy suite turnover rates in an outpatient setting. Furthermore, favorable patient evaluations suggest that sedation-assisted endoscopy can reduce patient anxiety and improve compliance with endoscopy procedures. Remimazolam is therefore expected to become the standard sedative for upper gastrointestinal endoscopy in outpatients in the future.

This study had several limitations. First, because the sample size was small, rare adverse events may have been undetected or not occurred. Furthermore, the small sample size may somewhat limit the strength of the results. Second, although patients were blinded, endoscopists were not; such a single-blind design might have caused bias for the decision of additional sedation by the endoscopists to some extent. However, since it was a multicenter study conducted in real-world clinical settings, we considered that blinding of endoscopists was not feasible to ensure safe sedative administration and appropriate intra-procedural management. Third, because the trial focused on upper GI endoscopy, the findings may not be generalizable to longer therapeutic procedures or higher-risk cohorts (e.g., ASA-PS score ≥ III, older adults).

In conclusion, this randomized, multicenter, head-to-head trial demonstrated that remimazolam yielded substantially faster recovery and less frequent need for supplemental oxygen than midazolam, while maintaining comparable initial sedation success. These findings support the use of remimazolam as an effective and operationally advantageous benzodiazepine for sedated upper gastrointestinal endoscopy in Japan.

## Supplementary Information

Below is the link to the electronic supplementary material.

Supplementary file1 (PDF 114 KB)

Supplementary file2 (PDF 78 KB)

Supplementary file3 (DOCX 19 KB)

## Abbreviations

ASA-PS: American Society of Anesthesiologists physical status
MOAA/S: Modified Observer assessment of alertness/sedation
