# Supplementary figures and images for: Efficacy and recovery of remimazolam versus midazolam in sedated upper gastrointestinal endoscopy: a multicenter randomized controlled trial in Japan (RECOVER Study)
Source: J Gastroenterol. 2025 Nov 17;61(3):241–9. doi: 10.1007/s00535-025-02324-x (PMC12987776; doi:10.1007/s00535-025-02324-x)

## Supplemental Figure1

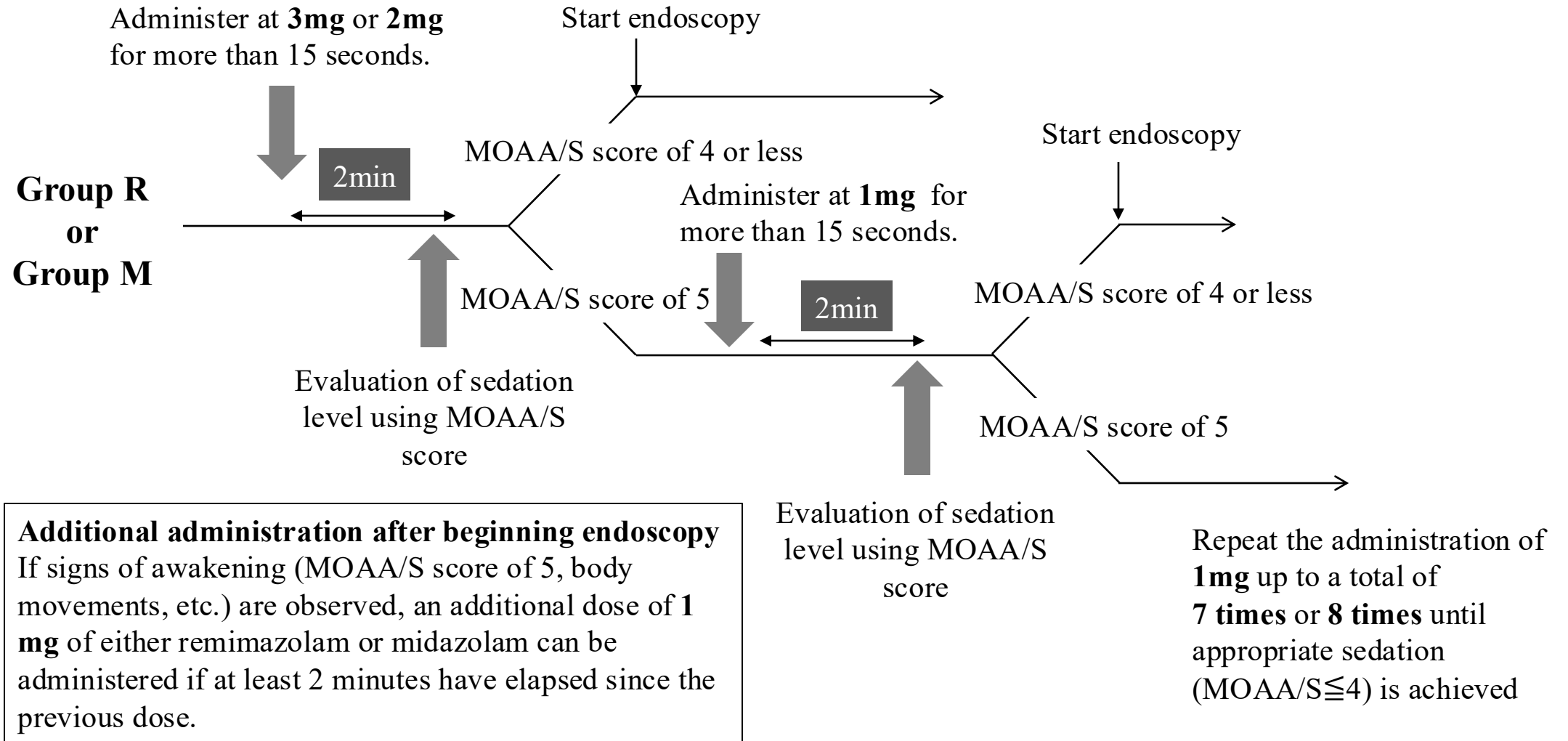

Supplement: Supplementary file 1 — Supplementary file1 (PDF 114 KB) [file 535_2025_2324_MOESM1_ESM.pdf]
