# Supplementary material for: Efficacy and recovery of remimazolam versus midazolam in sedated upper gastrointestinal endoscopy: a multicenter randomized controlled trial in Japan (RECOVER Study)
Source: J Gastroenterol. 2025 Nov 17;61(3):241–9. doi: 10.1007/s00535-025-02324-x (PMC12987776; doi:10.1007/s00535-025-02324-x)
Supplement: Supplementary file 2 — Supplementary file2 (PDF 78 KB) [file 535_2025_2324_MOESM2_ESM.pdf]

Supplemental Figure 2

MOAA/S	Score Description
5	Responds readily to name spoken in normal tone
4	Lethargic response to name spoken in normal tone
3	Responds only after name is called loudly and/or repeatedly
2	Responds only after mild prodding or shaking
1	Responds only after painful trapezius squeeze
0	No response after painful trapezius squeeze
