# Supplementary material for: Efficacy and recovery of remimazolam versus midazolam in sedated upper gastrointestinal endoscopy: a multicenter randomized controlled trial in Japan (RECOVER Study)
Source: J Gastroenterol. 2025 Nov 17;61(3):241–9. doi: 10.1007/s00535-025-02324-x (PMC12987776; doi:10.1007/s00535-025-02324-x)
Supplement: Supplementary file 3 — Supplementary file3 (DOCX 19 KB) [file 535_2025_2324_MOESM3_ESM.docx]

**Supplementary Table 1.** Molecular weight of sedative dosage required for Upper GI Endoscopy

|  | Group | n | Mean | SD | Min–Max | Mean difference | [ 95% CI] | p-value |
| --- | --- | --- | --- | --- | --- | --- | --- | --- |
| Pre-endoscopy dosage | Remimazolam | 20 | 9.00 | 3.00 | 6.8–15.9 | -0.06 | [-1.94, 1.82] | 0.947 |
| (μmol) | Midazolam | 18 | 9.06 | 2.68 | 6.2–15.4 |  |  |  |
| During endoscopy dosage | Remimazolam | 20 | 0.80 | 1.34 | 0–4. 6 | 0.28 | [-0.55, 1.12] | 0.494 |
| (μmol) | Midazolam | 18 | 0.51 | 1.10 | 0–3.1 |  |  |  |
| Total dosage | Remimazolam | 20 | 9.79 | 3.06 | 6.8–15.9 | 0.22 | [-1.64, 2.09] | 0.811 |
| (μmol) | Midazolam | 18 | 9.57 | 2.56 | 6.2–15.4 |  |  |  |

Remimazolam = 439 g/mol, Midazolam = 325 g/mol

GI: gastrointestinal
